# The Effects of GH Transgenic Goats on the Microflora of the Intestine, Feces and Surrounding Soil

**DOI:** 10.1371/journal.pone.0139822

**Published:** 2015-10-07

**Authors:** Zekun Bao, Xue Gao, Qiang Zhang, Jian Lin, Weiwei Hu, Huiqing Yu, Jianquan Chen, Qian Yang, Qinghua Yu

**Affiliations:** 1 College of Veterinary Medicine, Nanjing Agricultural University, Nanjing, China; 2 Shanghai Transgenic Research Center, 88 Cai-Lun Road, Shanghai, 201210, People’s Republic of China; University of Connecticut, UNITED STATES

## Abstract

The development of genetically engineered animals has brought with it increasing concerns about biosafety issues. We therefore evaluated the risks of growth hormone from transgenic goats, including the probability of horizontal gene transfer and the impact on the microbial community of the goats’ gastrointestinal tracts, feces and the surrounding soil. The results showed that neither the *GH* nor the *neoR* gene could be detected in the samples. Moreover, there was no significant change in the microbial community of the gastrointestinal tracts, feces and soil, as tested with PCR-denaturing gradient gel electrophoresis and 16S rDNA sequencing. Finally, phylogenetic analysis showed that the intestinal content, feces and soil samples all contained the same dominant group of bacteria. These results demonstrated that expression of goat growth hormone in the mammary of *GH* transgenic goat does not influence the microflora of the intestine, feces and surrounding soil.

## Introduction

The breeding of animals using genetically engineered (GE) technology has recently become possible. This process could avoid time-consuming artificial hybridization breeding and pure breeding programs. To date, many GE animals have been produced, including fish [1], mice [1], rabbits [2], sheep [2], pigs [2, 3], cows [4], and goats [5]. However, the safety evaluation of GE animals and food products should be considered seriously, especially with regard to potential effects on microflora through possible horizontal gene transfer. Horizontal gene transfer, also known as lateral gene transfer, refers to the transfer of genes between different species, such as between prokaryotes and eukaryotes in a manner other than traditional reproduction [6]. According to former reports, this phenomenon can take place between different species, including between bacteria and bacteria, between plants and bacteria, and between animals and plants [7–10]. The microbial community of the gastrointestinal tract is closely associated with the host metabolism and has a complex and sensitive construction [11]. Microflora may thus be an important intermediate by which horizontal gene transfer reaches other more advanced organisms.

To date, horizontal gene transfer between GE animals and bacteria has not been reported. However, further evidence is required to investigate this issue given the significant concerns. It is important to determine whether the structure of gastrointestinal bacterial flora could be rearranged following the insertion of foreign genes into GE animals and their alteration of the host metabolism. Moreover, soil contains various types of bacteria, and the bacteria in the gastrointestinal tract can also enter the environmental soil in the form of feces produced by GE animals. Any changes in the gastrointestinal bacterial flora could thus conceivably also influence the surrounding environmental soil flora [12].

To enhance the milk production of goats, we previously generated transgenic goats over-expressing goat growth hormone (*GH*) with beta-lactoglobulin promoter in their mammary glands by somatic cell nuclear transfer (SCNT). *GH* transgenic goats were confirmed by PCR analysis and verified the transgenic copy number and integration sites [13, 14]. Here, we focused on the effects of the *GH* transgenic goat on the microflora of the intestine, feces and surrounding soil.

## Materials and Methods

### Ethics statement

This study was approved by the Ethical Committee of Animal Experiments of the College of Veterinary Medicine at Nanjing Agricultural University. All animal care and use procedures were conducted in strict accordance with the Animal Research Committee guidelines of the College of Veterinary Medicine at Nanjing Agricultural University. All sections of this experiment adhere to the ARRIVE Guidelines for reporting animal research [15]. A completed ARRIVE guidelines checklist is included in S1 Checklist.

### Experimental animals and sample methods

Female *GH* transgenic and non-transgenic Saanen dairy goats were raised on a farm in the Transgenic Research Center, Shanghai, China. All the goats were healthy and fed with the same fodder (Table 1). During the entire experimental period in autumn, the goats were given ad libitum access to feed and water. The room temperature was maintained at 25–27°C. During housing, all animals were monitored twice daily to assess their health status. No adverse events were observed. Feces were taken from GH transgenic and non-transgenic goats, and each sample was taken when it just been defecated. Soil samples were taken from 0 m to 150 m from the GH transgenic goats’ pen with 15m in width and 30m in length. We slaughtered the goats and cut the intestine lengthwise to collect the intestinal contents from jejunum and cecum. The feces and soil samples were collected in three replications and mixed in one centrifuge tube, and the intestinal contents samples were collected only once. The samples details were performed in Table 2. All the fresh samples are stored in -70°C before analysis.

**Table 1:** Detailed information about the goats.

No.^a^	Target gene^b^	Promoter^c^	Generation	Age (year) and Sex	Gene manipulation^d^
1	*GH*	beta-lactoglobulin	F0	3, female	SCNT
2	*GH*	beta-lactoglobulin	F0	3, female	SCNT
3	*GH*	beta-lactoglobulin	F0	3, female	SCNT
4	*GH*	beta-lactoglobulin	F0	3, female	SCNT
5	*GH*	beta-lactoglobulin	F1	2, female	Breeding
6	*GH*	beta-lactoglobulin	F1	2, female	Breeding
7	*GH*	beta-lactoglobulin	F1	2, female	Breeding
8	*GH*	beta-lactoglobulin	F2	1, female	Breeding
9	*GH*	beta-lactoglobulin	F2	1, female	Breeding
10	*GH*	beta-lactoglobulin	F2	1, female	Breeding
11–14	None	None	-	3, female	Control

^a^ 1–10: transgenic goats, 11–14: non-transgenic goats

^b^ *GH*: growth hormone

^c^ Our previous study provided the detailed sequence information

^d^ SCNT: somatic cell nuclear transfer.

**Table 2:** Feces, soil and intestinal content samples.

Feces			Soil			Intestinal content		
Sample	Goat	Note	Sample	Goat	Distance from GH goat pen (m)	Sample	Goat	Location
S1	42007	GH F0	S5	-	0	S9	4	Jejunum
S2	42131	GH F1	S6	-	50	S10	14	Jejunum
S3	42226	GH F2	S7	-	100	S11	4	Cecum
S4	42321	Control	S8	-	150	S12	14	Cecum

1–10: transgenic goats, 11–14: non-transgenic goats.

### DNA extraction and PCR detection of target DNA

Microbial community DNA extraction of the fecal and intestinal samples was performed using the TIANamp Stool DNA Kit (Tiangen, China). The soil microbial community DNA extraction was performed using the EZNA Soil DNA Kit (Omega, USA). PCR amplifications of the *GH* and *neoR* gene fragments from the feces, soil and intestinal content samples were carried out, with positive and blank controls included in all procedures. The primers used were *GH*-F CATCCAGAAGGAATTCATGATGGCT, *GH*-R AGGGTCGACCTAGAAGGCACAGCT, *neoR*-F CCTGTCATCTCACCTTGCTCCT and neoR-R ATACCTGTCCGCCTTTCTCCCT. The PCR amplifications were carried out in 20 μl reaction volumes comprised of 10 μl of 2 × Taq Master Mix, 1 μl of each primer (10 μM), 0.2 μg of temple DNA and added ddH_2_O to 20 μl. Each target gene was amplified with an initial denaturation of DNA at 94°C for 10 min, followed by 26 cycles of 30 s of denaturation at 94°C, 60 s annealing at 60°C and 60 s of elongation at 72°C, with a final elongation for 10 min at the same temperature. PCR products were visualized by electrophoresis.

### Polymerase chain reaction-denaturing gradient gel electrophoresis (PCR-DGGE) analysis

PCR amplification of the variable V3 region of bacterial 16S rDNA was performed with a pair of universal primers (338F 5’-ACTCCTACGGGAGGCAGCAG–3’ and 518R 5’-ATTACCGCGGCTGCTGG–3) with a GC clamp of 39 bases (CGCCCGCCGCGCGCGGCGGGCGGGGCGGGGGCACGGGGGG) added to the 5’-terminus. PCR amplification was carried out in 50 μl reaction volumes, composed of 5 μl of 10 × PCR buffer, 4 μl of dNTP mixture, 1 μl of each primer (20 μM), 0.25 μl rTaq polymerase (5 U/μl), 2.5 ng of temple DNA and ddH_2_O to 50 μl. The 16S rDNA genes were amplified with an initial denaturation of DNA at 94°C for 10 min, followed by 30 cycles of 60 s of denaturation at 94°C, 60 s annealing at 55°C, 90 s of elongation at 72°C, and a final elongation for 10 min at the same temperature. PCR products were subjected to DGGE analysis with the Dcode Universal Mutation Detection System (Bio-Rad, Hercules, USA). The PCR products were loaded onto 8% (wt/vol) polyacrylamide (37:1 acrylamide/bisacrylamide) gels in 1× TAE buffer with a denaturing gradient ranging from 30 to 60%. The gels were run at 150 V for 420 min and then silver stained.

### Cloning and sequencing

The PCR products of the 16S rDNA from prominent bands were recovered with Gel Recovery Purification kits (Watson, China) and ligated into the pMD19-T vector (Takara, China). Then, these recombinant plasmids were transformed into *Escherichia coli* DH5α. Three or five clones were randomly selected for each band. These products were sequenced with an ABI Prism Big Dye Terminator Cycle Sequencing Ready Kit (Applied Biosystems, USA) with a pair of universal primers for the pMD19-T vector: M13F (-40) GTTTCCCAGTCACGAC and M13R (-26) CAGGAAACAGCTATGAC.

### Phylogenetic analysis

The analysis of 16S rDNA gene sequences was performed using the database of the National Center for Biotechnology Information (NCBI) to acquire closely related sequences. Phylogenetic trees were constructed with MEGA 6.0 on the basis of 97% similarity by the bootstrapped neighbor-joining method with 1000 iterations (S1 Fig). The similarity in the microorganism was compared by cluster analysis. Cluster analysis was performed based on Dice’s algorithm [16] with the BioEdit 7.0, Phylip 4.0, and MEGA 4.0 software.

## Results

### Detection of the transgene and marker gene

**Fig 1:**
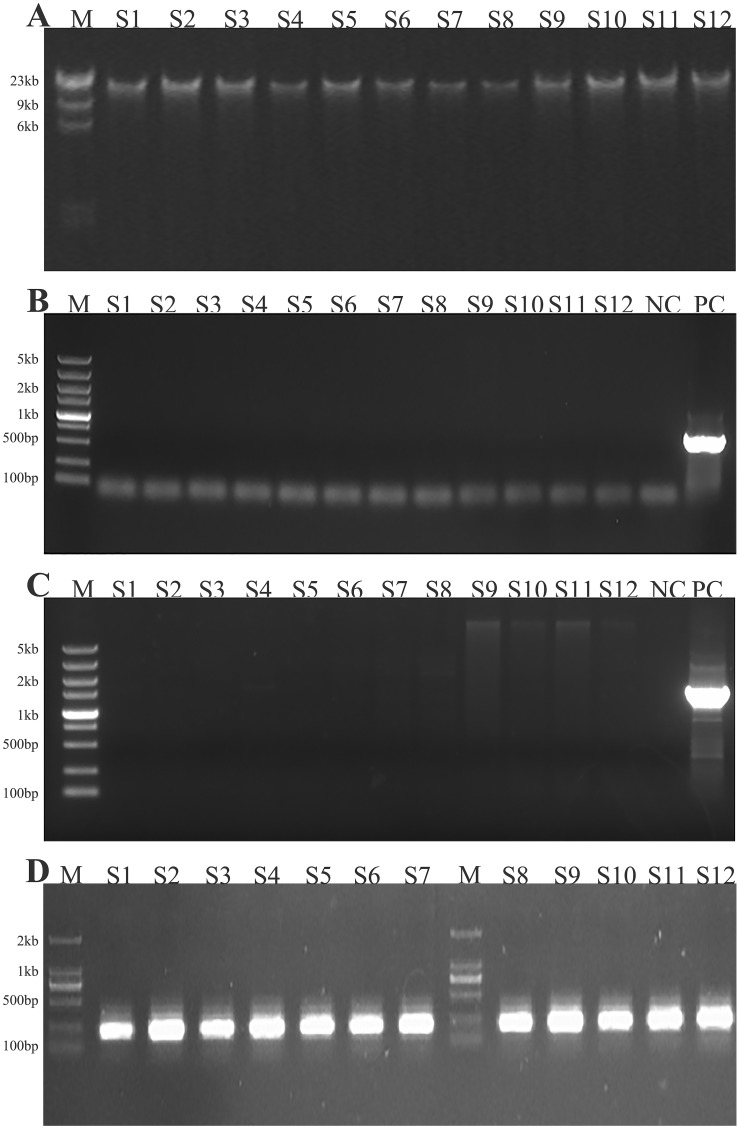
(A) Electrophoresis verification of the DNA extraction of samples. M: marker. (B) PCR results for the growth hormone gene detection experiment. NC: negative control with ddH_2_O. PC: positive control with pcGH vector over-expression. (C) PCR result of the *neoR* gene detection. NC: negative control with ddH_2_O. PC: positive control with over-expression of the pcGH vector. (D) PCR amplification of the 16 s rDNA of bacteria from samples.

The isolation of manure, soil, and intestinal contents DNA extraction were verified by electrophoresis (Fig 1A). Neither the transgene (*GH*) nor the marker gene (*neoR*) were detected in the feces, soil or intestinal content samples (Fig 1B and 1C). Bacterial genes were amplified with the 16S rDNA universal primers to verify the effectiveness of the DNA extraction (Fig 1D). The results showed that the extracted DNA from all samples contained the bacterial gene.

### PCR-DGGE and cluster analysis

**Fig 2:**
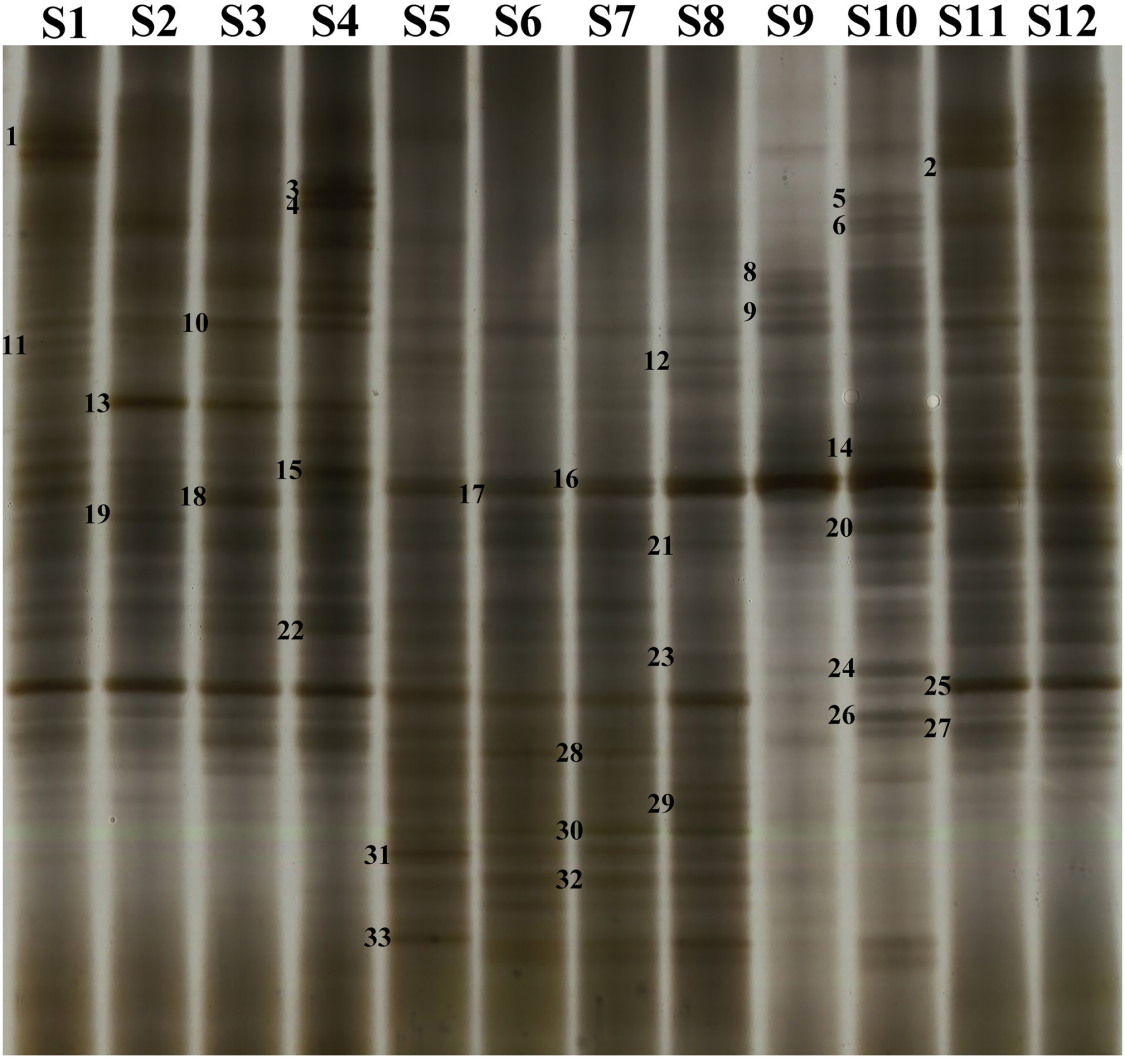
DGGE analysis of 16S rDNA fragments obtained after PCR amplification of the variable V3 region with universal primers 338F and 534R. The DGGE profiles for the total microbial DNAs extracted from samples are shown. The samples are described in Table 2. The numbers in the figures indicate the DGGE bands selected for cloning and sequencing.

**Fig 3:**
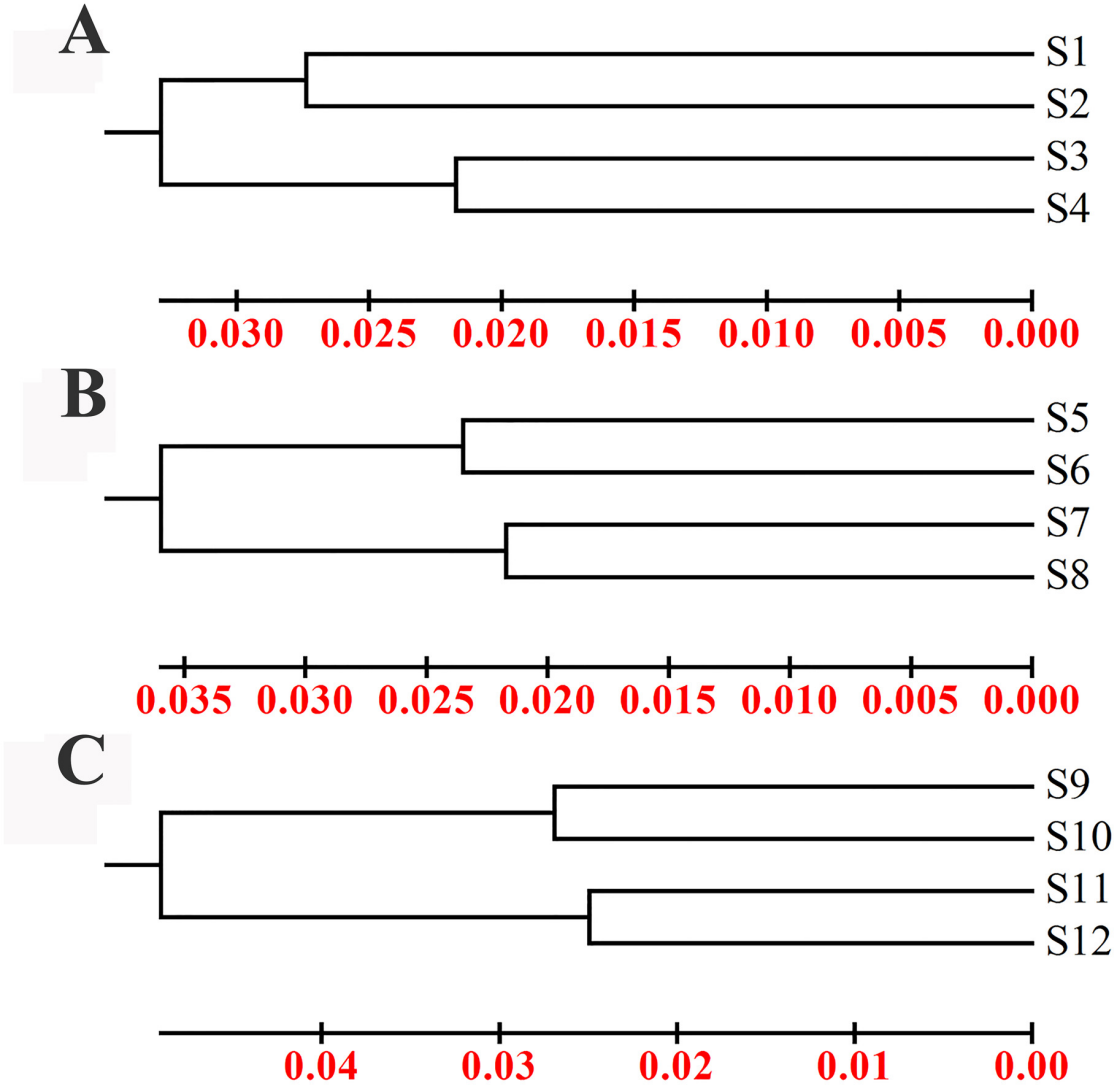
Cluster analysis based on the UPGMA of the DGGE profiles of the feces (A), soil (B) and intestinal content (C) samples. Scale bars indicate differences among the profiles.

To study the influence of the *GH* and *neoR* genes on the bacterial community structure, DGGE was performed after PCR amplification of the variable V3 region of the 16S rDNA from the microbial DNAs. Thirty-three distinct bands were found (Fig 2). Cluster analysis was also performed on the basis of similarity (> 95%) (Fig 3). The band patterns for the feces, soil and intestinal content samples showed degrees of similarity that were higher than 96%, 96.5% and 95%, respectively (Fig 3).

### Phylogenetic analysis

**Fig 4:**
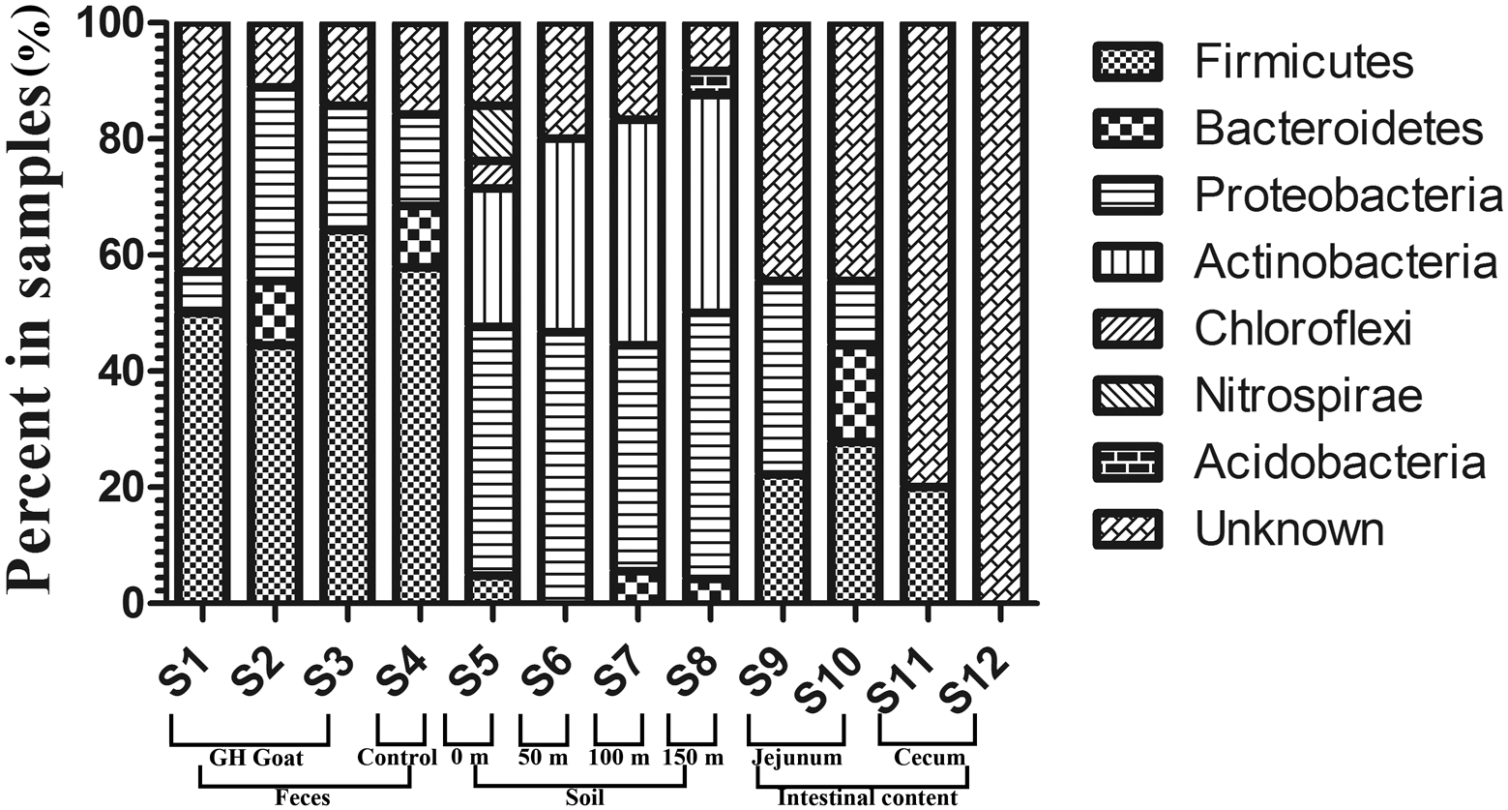
Phyla distribution of the 16S rDNA clone libraries obtained from the samples.

Each prominent DGGE band was recovered from the gels, cloned and sequenced. We selected sequences with over 97% similarity for subsequent phylogenetic analysis (S1 Table). Seven groups could be found based on the phylogenetic distribution of the 16S rDNA cloning libraries: *Firmicutes*, *Bacteroidetes*, *Proteobacteria*, *Acitinobacteria*, *Chloroflexi*, *Nitrospirae* and *Acidobacteria*. Unclassified sequences were designated as “unknown” (Fig 4). A more detailed classification is provided in S2 Fig.

## Discussion

Most GE research to date has focused on the animal health [16] and welfare, while the environmental assessment of GE animals is only beginning to be investigated. The intestinal flora of mature livestock should be stable. However, diverse species of bacteria colonize the GI tract and they may develop natural competence, or the ability to absorb naked DNA [6]. It is therefore necessary to detect bacterial changes in the feces of GE livestock in any complete environmental assessment. This is the first study of transgenic goats studied on their fecal matters and environment. We collected samples from the intestinal content, feces and soil. The GE goats were raised in a farm isolated to prevent communication with external wildlife and the escape of GE goats. We minimized the risk of horizontal gene transfer in the GE goats, although it still could occur in the gut and rumen. No *GH* and *neoR* were detected in the intestinal content, feces and soil samples, suggesting that no horizontal gene transfer occurred in the course of this study.

The microbial community of the gut can be affected by many factors, including animal health [17], age [18] and foraging patterns [19]. We minimized the influences of these factors in this study by selecting goats with the same rearing condition, of the same developmental stage, and foraging in the same location. PCR-DGGE and 16S rDNA sequencing were used to determine the genetic diversity of their microbial communities and to identify several uncultured microorganisms. The V3 region was selected for species identification, which can be used to distinguish bacterial species to the genus level [20]. Cluster analysis of intestinal contents and feces on the basis of 95% similarity showed that the microflora between the transgenic goats and normal goats were similar. Similar results were also detected in the soil samples, while three soil samples (S5, S6, and S7) covered the range of the farm, and S8 was collected from the outside of the farm. Furthermore, the phyla distribution among the three generations of GE goats (F0, F1 and F2) and normal goats was in the same dominant group, which could be because the microbial flora formed after weaning, then became stable.

In conclusion, we did not find any evidence of horizontal gene transfer from the *GH* transgenic goats to the gut floral of other goats or soil microorganisms. We also demonstrated that the foreign *GH* gene and *neoR* gene did not change the microbial flora in the goat intestine or the surrounding soil.

## Supporting Information

S1 Checklist NC3Rs ARRIVE Guidelines Checklist. (DOCX)

S1 Fig Neighbor-joining dendrogram derived from the 16S rRNA gene sequences (V3 region) of the predominant bands in the DGGE gels. Bootstrap confidence levels greater than 50% are indicated at the nodes (replicate 1,000 times). The scale bar indicates 2% divergence. For each tree entry in this study, the number before the hyphen represents the band excised from DGGE gels, and the number after the hyphen represents the clone from that band.
(PDF)

S2 Fig Identification of the clone alignment from NCBI. The number at the head of each paragraph is the sequencing clone, while the number at the end of each paragraph is the classification from the kingdom to the species.
(PDF)

S1 Table Sequence alignment of the bands from the DGGE gel. (PDF)

## Abbreviations

GE: Genetically engineered
*GH*: Growth hormone
SCNT: Somatic cell nuclear transfer
*neoR*: Neomycin resistance gene
