# Supplementary material for: The Effects of GH Transgenic Goats on the Microflora of the Intestine, Feces and Surrounding Soil
Source: PLoS One. 2015 Oct 7;10(10):e0139822. doi: 10.1371/journal.pone.0139822 (PMC4596820; doi:10.1371/journal.pone.0139822)
Supplement: S1 Fig — Bootstrap confidence levels greater than 50% are indicated at the nodes (replicate 1,000 times). The scale bar indicates 2% divergence. For each tree entry in this study, the number before the hyphen represents the band excised from DGGE gels, and the number after the hyphen represents the clone from that band. (PDF) [file pone.0139822.s002.pdf]

Figure S1

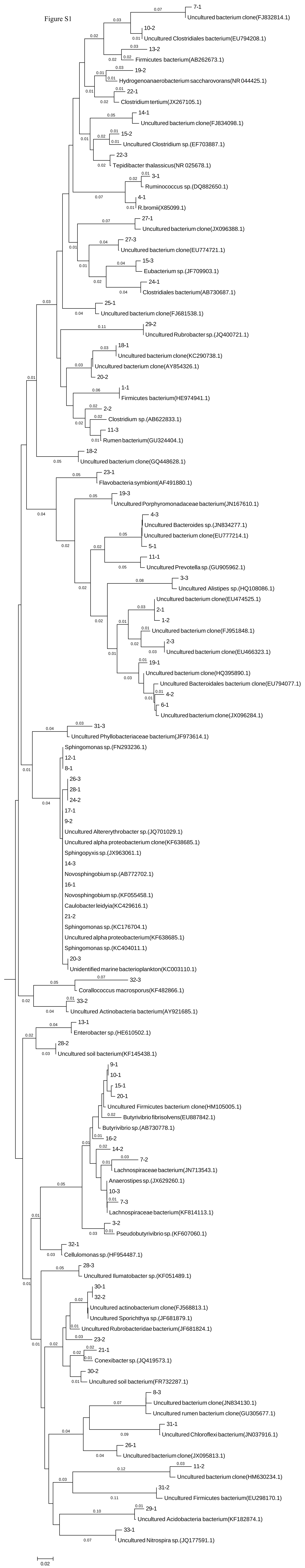

7-1
Uncultured bacterium clone(FJ832814.1)
10-2
Uncultured Clostridiales bacterium(EU794208.1)
13-2
Firmicutes bacterium(AB262673.1)
19-2
Hydrogenoanaerobacterium saccharovorans(NR 044425.1)
22-1
Clostridium tertium(JX267105.1)
14-1
Uncultured bacterium clone(FJ834098.1)
15-2
Uncultured Clostridium sp.(EF703887.1)
22-3
Tepidibacter thalassicus(NR 025678.1)
3-1
Ruminococcus sp.(DQ882650.1)
4-1
R.bromii(X85099.1)
27-1
Uncultured bacterium clone(JX096388.1)
27-3
Uncultured bacterium clone(EU774721.1)
15-3
Eubacterium sp.(JF709903.1)
24-1
Clostridiales bacterium(AB730687.1)
25-1
Uncultured bacterium clone(FJ681538.1)
29-2
Uncultured Rubrobacter sp.(JQ400721.1)
18-1
Uncultured bacterium clone(KC290738.1)
Uncultured bacterium clone(AY854326.1)
20-2
1-1
Firmicutes bacterium(HE974941.1)
2-2
Clostridium sp.(AB622833.1)
11-3
Rumen bacterium(GU324404.1)
18-2
Uncultured bacterium clone(GQ448628.1)
23-1
Flavobacteria symbiont(AF491880.1)
19-3
Uncultured Porphyromonadaceae bacterium(JN167610.1)
4-3
Uncultured Bacteroides sp.(JN834277.1)
Uncultured bacterium clone(EU777214.1)
5-1
11-1
Uncultured Prevotella sp.(GU905962.1)
3-3
Uncultured Alistipes sp.(HQ108086.1)
Uncultured bacterium clone(EU474525.1)
2-1
1-2
Uncultured bacterium clone(FJ951848.1)
2-3
Uncultured bacterium clone(EU466323.1)
19-1
Uncultured bacterium clone(HQ395890.1)
Uncultured Bacteroidales bacterium clone(EU794077.1)
4-2
6-1
Uncultured bacterium clone(JX096284.1)
31-3
Uncultured Phyllobacteriaceae bacterium(JF973614.1)
Sphingomonas sp.(FN293236.1)
12-1
8-1
26-3
28-1
24-2
17-1
9-2
Uncultured Altererythrobacter sp.(JQ701029.1)
Uncultured alpha proteobacterium clone(KF638685.1)
Sphingopyxis sp.(JX963061.1)
14-3
Novosphingobium sp.(AB772702.1)
16-1
Novosphingobium sp.(KF055458.1)
Caulobacter leidyia(KC429616.1)
21-2
Sphingomonas sp.(KC176704.1)
Uncultured alpha proteobacterium(KF638685.1)
Sphingomonas sp.(KC404011.1)
20-3
Unidentified marine bacterioplankton(KC003110.1)
32-3
Corallococcus macrosporus(KF482866.1)
33-2
Uncultured Actinobacteria bacterium(AY921685.1)
13-1
Enterobacter sp.(HE610502.1)
28-2
Uncultured soil bacterium(KF145438.1)
9-1
10-1
15-1
20-1
Uncultured Firmicutes bacterium clone(HM105005.1)
Butyrivibrio fibrisolvens(EU887842.1)
Butyrivibrio sp.(AB730778.1)
16-2
14-2
7-2
Lachnospiraceae bacterium(JN713543.1)
Anaerostipes sp.(JX629260.1)
10-3
7-3
Lachnospiraceae bacterium(KF814113.1)
3-2
Pseudobutyrivibrio sp.(KF607060.1)
32-1
Cellulomonas sp.(HF954487.1)
28-3
Uncultured Ilumatobacter sp.(KF051489.1)
30-1
32-2
Uncultured actinobacterium clone(FJ568813.1)
Uncultured Sporichthya sp.(JF681879.1)
Uncultured Rubrobacteridae bacterium(JF681824.1)
23-2
21-1
Conexibacter sp.(JQ419573.1)
30-2
Uncultured soil bacterium(FR732287.1)
8-3
Uncultured bacterium clone(JN834130.1)
Uncultured rumen bacterium clone(GU305677.1)
31-1
Uncultured Chloroflexi bacterium(JN037916.1)
26-1
Uncultured bacterium clone(JX095813.1)
11-2
Uncultured bacterium clone(HM630234.1)
31-2
Uncultured Firmicutes bacterium(EU298170.1)
29-1
Uncultured Acidobacteria bacterium(KF182874.1)
33-1
Uncultured Nitrospira sp.(JQ177591.1)
0.02

Figure **S1**. Neighbor-joining dendrogram derived from 16S rRNA gene sequences (V3 region) of predominet bands in DGGE gels. Bootstrap confidence levles grater than 50% are indicated at the nodes (replicate 1,000 times). The scale bar indicates 2% divergence. For each tree entry from this study, the number ahead of the hyphen represents the band excised from DGGE gels, and the number behind the hyphen represents the clone from that band.
