# Supplementary material for: The Effects of GH Transgenic Goats on the Microflora of the Intestine, Feces and Surrounding Soil
Source: PLoS One. 2015 Oct 7;10(10):e0139822. doi: 10.1371/journal.pone.0139822 (PMC4596820; doi:10.1371/journal.pone.0139822)
Supplement: S2 Fig — The number at the head of each paragraph is the sequencing clone, while the number at the end of each paragraph is the classification from the kingdom to the species. (PDF) [file pone.0139822.s003.pdf]

1-1 Bacteria; Firmicutes.
1-2 Bacteria; environmental samples.
2-1 Bacteria; environmental samples.
2-2 Bacteria; Firmicutes; Clostridia; Clostridiales; Clostridiaceae; Clostridium.
2-3 Bacteria; environmental samples.
3-1 Bacteria; Firmicutes; Clostridia; Clostridiales; Ruminococcaceae; Ruminococcus.
3-2 Bacteria; Firmicutes; Clostridia; Clostridiales; Lachnospiraceae; Pseudobutyrivibrio.
3-3 Bacteria; Bacteroidetes; Bacteroidia; Bacteroidales; Rikenellaceae; Alistipes; environmental samples.
4-1 Bacteria; Firmicutes; Clostridia; Clostridiales; Ruminococcaceae; Ruminococcus.
4-2 Bacteria; Bacteroidetes; Bacteroidia; Bacteroidales; environmental samples
4-3 Bacteria; environmental samples.
5-1 Bacteria; Bacteroidetes; Bacteroidia; Bacteroidales; Bacteroidaceae; Bacteroides; environmental samples.
6-1 Bacteria; environmental samples.
7-1 Bacteria; environmental samples
7-2 Bacteria; Firmicutes; Clostridia; Clostridiales; Lachnospiraceae.
7-3 Bacteria; Firmicutes; Clostridia; Clostridiales; Lachnospiraceae; Anaerostipes.
8-1 Bacteria; Proteobacteria; Alphaproteobacteria; Sphingomonadales; Sphingomonadaceae; Sphingomonas.
8-3 Bacteria; environmental samples.
9-1 Bacteria; environmental samples.
9-2 Bacteria; Proteobacteria; Alphaproteobacteria; Sphingomonadales; Erythrobacteraceae; Altererythrobacter; environmental samples.
10-1 Bacteria; Proteobacteria; Alphaproteobacteria; environmental samples.
10-2 Bacteria; Firmicutes; Clostridia; Clostridiales; environmental samples.
10-3 Bacteria; Firmicutes; Clostridia; Clostridiales; Lachnospiraceae.
11-1 Bacteria; Bacteroidetes; Bacteroidia; Bacteroidales;Prevotellaceae; Prevotella; environmental samples.
11-2 Bacteria; environmental samples.
11-3 Bacteria.
12-1 Bacteria; Proteobacteria; Alphaproteobacteria; Sphingomonadales; Sphingomonadaceae; Sphingopyxis; environmental samples.
13-1 Bacteria; Proteobacteria; Gammaproteobacteria; Enterobacteriales; Enterobacteriaceae; Enterobacter.
13-2 Bacteria; Firmicutes.
14-1 Bacteria; environmental samples.
14-2 Bacteria; Firmicutes; Clostridia; Clostridiales; Lachnospiraceae; Anaerostipes.
14-3 Bacteria; Proteobacteria; Alphaproteobacteria; Sphingomonadales; Sphingomonadaceae; Novosphingobium.
15-1 Bacteria; Firmicutes; Clostridia; Clostridiales; Lachnospiraceae; Butyrivibrio.
15-2 Bacteria; Firmicutes; Clostridia; Clostridiales; Clostridiaceae; Clostridium; environmental samples.
15-3 Bacteria; Firmicutes; Clostridia; Clostridiales; Eubacteriaceae; Eubacterium.

16-1 Bacteria; Proteobacteria; Alphaproteobacteria; Sphingomonadales; Sphingomonadaceae; Novosphingobium.
16-2 Bacteria; Firmicutes; environmental samples.
17-1 Bacteria; Proteobacteria; Alphaproteobacteria; Sphingomonadales; Sphingomonadaceae; Sphingomonas.
18-1 Bacteria; environmental samples.
18-2 Bacteria; environmental samples.
19-1 Bacteria; environmental samples.
19-2 Bacteria; Firmicutes; Clostridia; Clostridiales; Ruminococcaceae; Hydrogenoanaerobacterium.
19-3 Bacteria; Bacteroidetes; Bacteroidia; Bacteroidales; Porphyromonadaceae; environmental samples.
20-1 Bacteria; Firmicutes; Clostridia; Clostridiales; Lachnospiraceae; Butyrivibrio.
20-2 Bacteria; environmental samples.
20-3 Bacteria; environmental samples.
21-1 Bacteria; Actinobacteria; Rubrobacteridae; Solirubrobacterales; Conexibacteraceae; Conexibacter.
21-2 Bacteria; Proteobacteria; Alphaproteobacteria; Sphingomonadales; Sphingomonadaceae; Sphingomonas.
22-1 Bacteria; Firmicutes; Clostridia; Clostridiales; Clostridiaceae; Clostridium.
22-3 Bacteria; Firmicutes; Clostridia; Clostridiales; Peptostreptococcaceae; Tepidibacter.
23-1 Bacteria; Bacteroidetes; Flavobacteriia.
23-2 Bacteria; Actinobacteria; Rubrobacteridae; environmental samples.
24-1 Bacteria; Firmicutes; Clostridia; Clostridiales.
24-2 Bacteria; Proteobacteria; Alphaproteobacteria; environmental samples.
25-1 Bacteria; environmental samples.
26-1 Bacteria; environmental samples.
26-3 Bacteria; Proteobacteria; Alphaproteobacteria; environmental samples.
27-1 Bacteria; environmental samples.
27-3 Bacteria; environmental samples.
28-1 Bacteria; Proteobacteria; Alphaproteobacteria; Sphingomonadales; Sphingomonadaceae; Sphingomonas; environmental samples.
28-2 Bacteria; environmental samples.
28-3 Bacteria; Actinobacteria; Acidimicrobidae; Acidimicrobiales; Acidimicrobineae; Acidimicrobiaceae; Ilumatobacter; environmental samples.
29-1 Bacteria; Acidobacteria; environmental samples.
29-2 Bacteria; Actinobacteria; Rubrobacteridae; Rubrobacterales; Rubrobacterineae; Rubrobacteraceae; Rubrobacter; environmental samples.
30-1 Bacteria; Actinobacteria; environmental samples.
30-2 Bacteria; environmental samples.
31-1 Bacteria; Chloroflexi; environmental samples.
31-2 Bacteria; Firmicutes; environmental samples.
31-3 Bacteria; Proteobacteria; Alphaproteobacteria; Rhizobiales; Phyllobacteriaceae; environmental samples.

32-1 Bacteria; Actinobacteria; Actinobacteridae; Actinomycetales; Micrococcineae; Cellulomonadaceae; Cellulomonas.

32-2 Bacteria; Actinobacteria; Actinobacteridae; Actinomycetales; Frankineae; Sporichthyaceae; Sporichthya; environmental samples.

32-3 Bacteria; Proteobacteria; Deltaproteobacteria; Myxococcales; Cystobacterineae; Myxococcaceae; Corallococcus.

33-1 Bacteria; Nitrospirae; Nitrospirales; Nitrospiraceae; Nitrospira; environmental samples.

33-2 Bacteria; Actinobacteria; environmental samples.
