# Supplementary material for: The Effects of GH Transgenic Goats on the Microflora of the Intestine, Feces and Surrounding Soil
Source: PLoS One. 2015 Oct 7;10(10):e0139822. doi: 10.1371/journal.pone.0139822 (PMC4596820; doi:10.1371/journal.pone.0139822)
Supplement: S1 Table — (PDF) [file pone.0139822.s004.pdf]

S1 Table. Sequence alignment of the bands from DGGE gel

Band No.[a]	Clones No.[b]	Similarity[c]	NCBI alignment results[d]
1	1-1 (KP968279)	99%	Firmicutes bacterium(HE974941.1)
	1-2 (KP968280)	98%	Uncultured bacterium clone(EU474525.1)
2	2-1 (KP968281)	94%	Uncultured bacterium clone(FJ951848.1)
	2-2 (KP968282)	96%	Clostridium sp.(AB622833.1)
	2-3 (KP968283)	100%	Uncultured bacterium clone(EU466323.1)
3	3-1 (KP968284)	99%	Ruminococcus sp.(DQ882650.1)
	3-2 (KP968285)	98%	Pseudobutyrivibrio sp.(KF607060.1)
	3-3 (KP968286)	98%	Uncultured Alistipes sp.(HQ108086.1)
4	4-1(KP968287)	99%	R.bromii(X85099.1)
	4-2 (KP968288)	98%	Uncultured Bacteroidales bacterium clone(EU794077.1)
	4-3 (KP968289)	99%	Uncultured bacterium clone(EU777214.1)
5	5-1 (KP968290)	98%	Uncultured Bacteroides sp.(JN834277.1)
6	6-1 (KP968294)	99%	Uncultured bacterium clone(JX096284.1)
7	7-1 (KP968297)	99%	Uncultured bacterium clone(FJ832814.1)
	7-2 (KP968298)	97%	Lachnospiraceae bacterium(JN713543.1)
	7-3 (KP968299)	99%	Anaerostipes sp.(JX629260.1)
8	8-1 (KP968300)	100%	Sphingomonas sp.(FN293236.1)
	8-3 (KP968297)	99%	Uncultured rumen bacterium clone(GU305677.1)
9	9-1 (KP968303)	98%	Uncultured bacterium clone(JN834130.1)
	9-2 (KP968304)	100%	Uncultured Altererythrobacter sp.(JQ701029.1)
10	10-1 (KP968306)	100%	Uncultured alpha proteobacterium clone(KF638685.1)
	10-2 (KP968307)	98%	Uncultured Clostridiales bacterium(EU794208.1)
	10-3 (KP968308)	100%	Lachnospiraceae bacterium(KF814113.1)
11	11-1 (KP968309)	96%	Uncultured Prevotella sp.(GU905962.1)
	11-2 (KP968310)	96%	Uncultured bacterium clone(HM630234.1)
	11-3 (KP968311)	99%	Rumen bacterium(GU324404.1)

12	12-1 (KP968312)	99%	Sphingopyxis sp.(JX963061.1)
13	13-1 (KP968315)	99%	Enterobacter sp.(HE610502.1)
	13-2 (KP968316)	96%	Firmicutes bacterium(AB262673.1)
14	14-1 (KP968317)	98%	Uncultured bacterium clone(FJ834098.1)
	14-2 (KP968318)	98%	Anaerostipes sp.(JX629260.1)
	14-3 (KP968319)	100%	Novosphingobium sp.(AB772702.1)
15	15-2 (KP968320)	96%	Uncultured Clostridium sp.(EF703887.1)
	15-3 (KP968321)	98%	Eubacterium sp.(JF709903.1)
16	16-1 (KP968322)	100%	Novosphingobium sp.(KF055458.1)
	16-2 (KP968323)	99%	Uncultured Firmicutes bacterium clone(HM105005.1)
17	17-1 (KP968324)	100%	Caulobacter leidyia(KC429616.1)
18	18-1 (KP968327)	100%	Uncultured bacterium clone(KC290738.1)
	18-2 (KP968328)	99%	Uncultured bacterium clone(GQ448628.1)
19	19-1 (KP968330)	97%	Uncultured bacterium clone(HQ395890.1)
	19-2 (KP968331)	95%	Hydrogenoanaerobacterium saccharovorans(NR_044425.1)
	19-3 (KP968332)	97%	Uncultured Porphyromonadaceae bacterium(JN167610.1)
20	20-1 (KP968333)	97%	Butyrivibrio fibrisolvens(EU887842.1)
	20-2 (KP968334)	99%	Uncultured bacterium clone(AY854326.1)
	20-3 (KP968335)	100%	Unidentified marine bacterioplankton(KC003110.1)
21	21-1 (KP968336)	95%	Conexibacter sp.(JQ419573.1)
	21-2 (KP968337)	100%	Sphingomonas sp.(KC176704.1)

22	22-1 (KP968339)	98%	Clostridium tertium(JX267105.1)
	22-3 (KP968341)	98%	Tepidibacter thalassicus(NR_025678.1)
23	23-1 (KP968342)	99%	Flavobacteria symbiont(AF491880.1)
	23-2 (KP968343)	97%	Uncultured Rubrobacteridae bacterium(JF681824.1)
24	24-1 (KP968345)	99%	Clostridiales bacterium(AB730687.1)
	24-2 (KP968346)	99%	Uncultured alpha proteobacterium(KF638685.1)
25	25-1 (KP968348)	99%	Uncultured bacterium clone(FJ681538.1)
26	26-1 (KP968351)	99%	Uncultured bacterium clone(JX095813.1)
	26-3 (KP968353)	99%	Uncultured alpha proteobacterium(KF638685.1)
28	28-1 (KP968354)	99%	Sphingomonas sp.(KC404011.1)
	28-3 (KP968355)	99%	Uncultured Ilumatobacter sp.(KF051489.1)
29	29-1 (KP968356)	98%	Uncultured Acidobacteria bacterium(KF182874.1)
	29-2 (KP968357)	100%	Uncultured Rubrobacter sp.(JQ400721.1)
30	30-1 (KP968359)	99%	Uncultured actinobacterium clone(FJ568813.1)
	30-2 (KP968360)	99%	Uncultured soil bacterium(FR732287.1)
31	31-1 (KP968361)	99%	Uncultured Chloroflexi bacterium(JN037916.1)
	31-2 (KP968362)	99%	Uncultured Firmicutes bacterium(EU298170.1)
	31-3 (KP968363)	97%	Uncultured Phyllobacteriaceae bacterium(JF973614.1)
32	32-1 (KP968364)	99%	Cellulomonas sp.(HF954487.1)
	32-2 (KP968365)	99%	Uncultured Sporichthya sp.(JF681879.1)
33	33-1 (KP968366)	99%	Uncultured Nitrospira sp.(JQ177591.1)
	33-2 (KP968367)	99%	Uncultured Actinobacteria bacterium(AY921685.1)

[a] Number of the band in Fig. 2.
[b] Clone number of each band and Genbank accession number.
[c] Similarity between sequencing results and NCBI alignment results.
[d] Alignment results in NCBI.
